# Systematic profiling of alternative splicing events and splicing factors in left- and right-sided colon cancer

**DOI:** 10.18632/aging.102319

**Published:** 2019-10-04

**Authors:** Xiaoliang Huang, Jungang Liu, Xianwei Mo, Haizhou Liu, Chunyin Wei, Lingxu Huang, Jianhong Chen, Chao Tian, Yongsheng Meng, Guo Wu, Weishun Xie, Franco Jeen P.C., Zujun Liu, Weizhong Tang

**Affiliations:** 1 Department of Gastrointestinal Surgery, Guangxi Medical University Cancer Hospital, Nanning 530021, Guangxi Zhuang Autonomous Region, The People’s Republic of China; 2 Guangxi Clinical Research Center for Colorectal Cancer, Nanning 530021, Guangxi Zhuang Autonomous Region, The People’s Republic of China; 3 Department of Research, Guangxi Medical University Cancer Hospital, Nanning 530021, Guangxi Zhuang Autonomous Region, The People’s Republic of China

**Keywords:** FIP1L1, prognostic signature, splicing factor, SATB2, TCGA

## Abstract

Left- and right-sided colon cancer (LC and RC) differ substantially in their molecular characteristics and prognoses, and are thus treated using different strategies. We systematically analyzed alternative splicing (AS) events and splicing factors in LC and RC. RNA-seq data were used for genome-wide profiling of AS events that could distinguish LC from RC. The Exon Skip splicing pattern was more common in RC, while the Retained Intron pattern was more common in LC. The AS events that were upregulated in RC were enriched for genes in the axon guidance pathway, while those that were upregulated in LC were enriched for genes in immune-related pathways. Prognostic models based on differentially expressed AS events were built, and a prognostic signature based on these AS events performed well for risk stratification in colon cancer patients. A correlation network of differentially expressed AS events and differentially expressed splicing factors was constructed, and *RBM25* was identified as the hub gene in the network. In conclusion, large differences in AS events may contribute to the phenotypic differences between LC and RC. The differentially expressed AS events reported herein could be used as biomarkers and treatment targets for colon cancer.

## INTRODUCTION

Colorectal cancer (CRC) ranks as the third most frequently diagnosed type of cancer and the fourth leading cause of cancer-related death worldwide. It is estimated that over 2.2 million new cases and 1.1 million deaths will occur by 2030 [1]. Colon cancer is a heterogeneous disease. According to its anatomic site, colon cancer can be classified as left- or right-sided colon cancer (LC or RC), which are regarded as distinct diseases [2, 3]. The left and right sides of the colon have different embryonic origins: the left side is derived from the embryonic hindgut, while the right side is derived from the embryonic midgut [4]. These differences in origin contribute to biological differences between the left and right sides of the colon. Thus, LC and RC differ substantially in their pathogeneses, molecular characteristics, incidences and prognoses, and are treated by different strategies [4, 5].

In terms of the pathway of carcinogenesis, LC more frequently exhibits chromosomal instability, while RC more often displays microsatellite instability and cytosine-guanosine island hypermethylation [6, 7]. In addition, *APC* and *TP53* mutations are more prevalent in LC, while *BRAF* mutations are significantly more common in RC [8, 9]. The distribution of the four consensus molecular subtypes (CMSs) differs between LC and RC. LC is enriched in CMS2 (activation of the WNT and MYC pathways) and CMS4 (enhanced epithelial-mesenchymal transition), while RC is enriched in CMS1 (increased immune infiltration) and CMS3 (activation of multiple metabolic pathways) [10].

RC is more frequently found in female elderly patients, and is more likely to exhibit an undifferentiated or signet-ring-cell histology than LC [11]. The overall survival of RC patients is much poorer than that of LC patients [12]. Patients with RC have not benefited from first-line anti-EGFR (epidermal growth factor receptor)-based chemoimmunotherapy [4], while patients with RAS-wild-type metastatic LC receiving anti-EGFR-based chemoimmunotherapy have exhibited longer overall survival than those receiving anti-VEGF (vascular endothelial growth factor)-based chemoimmunotherapy [13]. Though the understanding of LC and RC is gradually deepening, much remains unknown concerning their molecular distinctions.

Alternative splicing (AS) is an RNA processing pathway in which a single pre-mRNA is spliced into different arrangements to produce structurally and functionally distinct mRNAs [14]. In the process of human gene expression, a gene is first transcribed into pre-mRNA, which contains an average of 8 to 10 coding exons separated by non-coding introns [15]. Then, the pre-mRNA is transformed into mRNA through the excision of introns and the ligation of exons. AS occurs when different exons or introns are retained or excluded to generate alternative mRNA transcripts [16], and this process significantly increases the proteome diversity and cell complexity [17]. AS explains why there are over 82,000 distinct mRNA sequences and around 2 million protein molecules in the human body, even though human cells only contain around 20,000 protein-coding genes [18, 19]. Up to 90% of human genes undergo AS [20].

AS profoundly alters the function of proteins by changing their stability, adding or deleting structural domains and modifying their protein-protein interactions [21]. AS has been increasingly implicated in human diseases, especially cancer [22]. The AS of genes modifies proteins involved in many malignant activities, including proliferation, invasion, metastasis, apoptosis, hypoxia, metabolic changes, angiogenesis and immune escape [23]. Aberrant AS is a potential biomarker of tumorigenesis and prognosis, and is also a therapeutic target in malignancy [24].

AS is orchestrated by a large and highly dynamic protein complex called the spliceosome [25], which recognizes and binds to pre-mRNAs at specific positions and subsequently processes them into mature RNAs [26]. The spliceosome consists of five small nuclear RNAs (snRNA U1, U2, U4, U5 and U6) and over 300 splicing factors (SFs) [16]. Among these SFs, two RNA SF families have been well-studied: the serine-arginine- rich SFs (SRSFs) and the heterogeneous nuclear ribonucleoproteins (HNRNPs) [27]. SRSFs tend to bind to intronic and exonic splicing enhancers, whereas HNRNPs mainly bind to exonic and intronic splicing silencers. Thus, SRSFs and HNRNPs are crucial for promoting exon skipping and exon inclusion, respectively. Abnormal SF expression and/or activity globally dysregulates AS events [28], and SFs may contribute to tumorigenesis as oncogenes or pseudo-oncogenes. Thus, it would be of great significance to draw a regulatory network that comprehensively describes the involvement of SFs in AS.

Several studies have identified cancer-specific AS events by comparing cancer patients with normal controls [29–31]. In CRC, 421 differentially expressed AS events (DEAS) were found, and the parent genes were enriched in protein kinase activity, phosphoinositide 3-kinase/Akt signaling and P53 signaling pathways [31]. However, to the best of our knowledge, the AS events and SFs in LC and RC have not been systematically compared, although such a comparison is greatly needed in view of the heterogeneity of colon cancer. To fill this gap, we systematically profiled the distinct AS events and SFs between LC and RC and built an interaction network from them. We also identified a series of distinct prognostic AS events and used them to construct a highly efficient prognostic signature.

## RESULTS

### Clinical features of left- and right-sided colon cancer patients

The present study included a total of 434 colon cancer patients, among whom 176 had LC (affecting the splenic flexure of the colon, descending colon and sigmoid colon) and 258 had RC (affecting the ileocecum, ascending colon, hepatic flexure of the colon and transverse colon). The clinical features of the LC and RC patients are listed in Table 1. The proportion of elderly patients was significantly higher in the RC group (50.8% vs. 35.2%, P=0.001). Distant and lymphatic metastases were significantly more common in LC patients (P<0.05). The proportion of patients with microsatellite instability was higher in the RC group than in the LC group (18.9% vs. 2.9%, P=0.064).

**Table 1:** Clinical features of left- and right-sided colon cancer patients.

**Features**	**Left-sided colon cancer(%) n=176**	**Right-sided colon cancer(%) n=258**	**P-values**
Age			0.001*
>70	62(35.2)	131(50.8)	
≤70	114(64.8)	127(49.2)	
Gender			0.90
Male	91(51.7)	135(52.3)	
Female	85(48.3)	123(47.7)	
BMI(kg/m^2)^			0.27
BMI <18.5	0(0.0)	1(0.7)	
18.5≤BMI<25	21(26.6)	50(36.8)	
25≤BMI<30	27(34.2)	45(33.1)	
30≤BMI	31(39.2)	40(29.4)	
Stage			0.067
I	29(16.7)	44(17.7)	
II	59(33.9)	109(43.8)	
III	53(30.5)	68(27.3)	
IV	33(19.0)	28(11.2)	
M category			0.035*
M1	33(20.6)	28(12.6)	
M0	127(79.4)	194(87.4)	
N category			0.032*
N1-2	83(47.2)	95(36.8)	
N0	93(52.8)	163(63.2)	
Venous invasion			0.52
Yes	40(26.0)	51(23.1)	
No	114(74.0)	170(76.9)	
Lymphatic invasion			0.64
Yes	72(45.3)	83(35.9)	
No	87(54.7)	148(64.1)	
Microsatellite instability			0.064
Yes	1(2.9)	10(18.9)	
No	33(97.1)	43(81.1)	
k-ras mutation			0.84
Yes	8(44.4)	10(47.6)	
No	10(55.6)	11(52.4)	
BRAF mutation			0.53
Yes	0(0.0)	3(17.6)	
No	8(100.0)	14(82.4)	

* P<0.05

### DEAS events in left- and right-sided colon cancer

**Figure 1:**
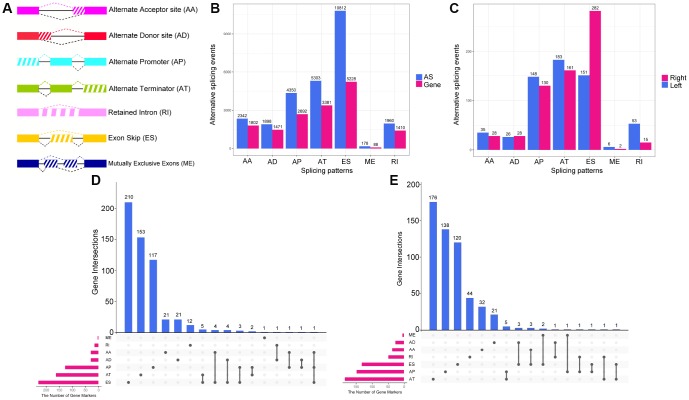
**Overview of AS event profiling in left- and right-sided colon cancer.** (**A**) Illustration of the seven types of AS events: Exon Skip (ES), Mutually Exclusive Exons (ME), Retained Intron (RI), Alternate Promoter (AP), Alternate Terminator (AT), Alternate Donor site (AD) and Alternate Acceptor site (AA). (**B**) The number of AS events and involved genes for each AS type in the 434 colon cancer patients. (**C**) The number of (DEAS events upregulated in left- or right-sided colon cancer. (**D**) UpSet plot of overlapping genes among the seven patterns of DEAS events that were upregulated in right-sided colon cancer. One gene may have up to three splicing patterns. (**E**) UpSet plot of overlapping genes among the seven patterns of DEAS events that were upregulated in left-sided colon cancer.

Integrated AS event profiling was performed with data from the 434 colon cancer patients. AS events were quantified based on their percent-spliced-in (PSI) values, which are commonly used for this purpose. We observed extremely low values (PSI <0.05) for certain splicing isoforms. To obtain a reliable a set of AS events in colon cancer, we only included AS events that occurred in at least 75% of the samples with an average PSI value ≥0.05. After the results were thus filtered, a total of 26843 AS events from 8879 genes remained for further analysis. Seven different splicing patterns were identified in colon cancer: Exon Skip (ES), Mutually Exclusive Exons (ME), Retained Intron (RI), Alternate Promoter (AP), Alternate Terminator (AT), Alternate Donor site (AD) and Alternate Acceptor site (AA) (Figure 1A). The ratio of AS events to genes was around 4:1, indicating that each gene underwent four AS events on average. ES was the most frequent splicing pattern, followed by AT and AP, while ME was the least frequent splicing pattern (Figure 1B).

To investigate the distinctions between LC and RC at the level of AS, we performed a differential expression analysis. Given the small range of PSI values (from zero to one), we filtered the results based on an adjusted P-value <0.05. Ultimately, 1248 DEAS from 836 genes were identified. Among the DEAS events, 646 AS events from 557 genes were upregulated in RC, while 602 AS events from 550 genes were upregulated in LC (Supplementary Figure 1). The proportion of different types of AS events differed significantly between LC and RC (Figure 1C, P=4.69×10^−12^); for instance, the ES pattern was significantly more common in RC, while the RI pattern was significantly more common in LC (P<0.05). Considering one gene had more than one AS pattern, we used UpSet plot to visualize the intersecting sets of different AS pattern. We found that, among the DEAS that were upregulated in RC, genes with the ES pattern occupied the largest number (210 cases), followed by those with the AT pattern and the AP pattern (Figure 1D). However, among the DEAS that were upregulated in LC, genes with the AT pattern occupied the largest number (176 cases), followed by those with the AP pattern and the ES pattern (Figure 1E). All these findings suggested that AS events contribute to the heterogeneity of LC and RC.

### Enrichment and interaction analysis of DEAS events

**Figure 2:**
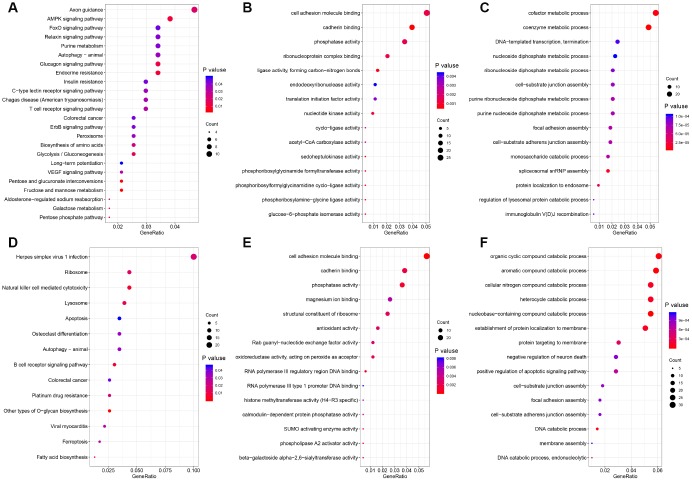
**Functional GO analysis and KEGG analysis of DSGs between left- and right-sided colon cancer.** The vertical axis represents GO or KEGG pathway annotations. The horizontal axis represents the number of genes assigned to the corresponding annotation. (**A**–**C**) right-sided colon cancer; (**D**–**F**) left-sided colon cancer. (**A** and **D**) KEGG pathways; (**B** and **E**) GO molecular functions; (**C** and **F**) GO biological processes.

AS can directly alter protein function. To explore the potential functions and pathways of the DEAS events, we performed an enrichment analysis of the differentially spliced genes (DSGs). As shown in Figure 2A, the DEAS that were upregulated in RC were significantly enriched for genes involved in axon guidance (P=0.02), adenosine monophosphate kinase signaling (P=0.009), FoxO signaling (P=0.04), VEGF signaling (P=0.03) and colorectal cancer (P=0.04). The DEAS that were upregulated in LC were significantly enriched for genes involved in B cell receptor signaling (P=0.004), natural killer cell-mediated cytotoxicity (P=0.005), ribosomes (P=0.01) and colorectal cancer (P=0.04) (Figure 2D). Gene ontology (GO) molecular function enrichment analysis indicated that cadherin binding (P=5.4×10^−3^) and cell adhesion molecule binding (P=1.3×10^−3^) were enriched in LC and RC, respectively (Figure 2B and 2E). GO biological process enrichment analysis indicated that coenzyme metabolic processes (P=5.1×10^−6^), cofactor metabolic processes (P=7.7×10^−6^) and spliceosomal small nuclear ribonucleoprotein assembly (P=1.2×10^−5^) were enriched in RC, while nucleobase-containing compound catabolic processes (P=1.3×10^−5^) and DNA catabolic processes (P=2.4×10^−5^) were enriched in LC (Figure 2C and 2F).

Since AS inevitably alters the translation and features of proteins, we performed a protein-protein interaction network analysis of the proteins encoded by the DSGs in LC and RC. Nodes with over 14 degrees were identified as hub genes with potentially vital regulatory functions in the network. The protein-protein interaction network of DSGs in LC is displayed in Supplementary Figure 2. There were 211 nodes and 405 edges in the network. *UBB*, *RNPS1*, *RPS29* and *PPP2R2A* were hub genes in the network. Supplementary Figure 3 depicts the protein-protein interaction network of DSGs in RC. There were 213 nodes and 389 edges in the network. Interestingly, most of the hub genes in RC encoded ribosomal proteins such as RPL37A, RPLP0, RPL24, RPL30 and RPL15, suggesting that the AS of genes encoding ribosomal proteins promotes the development of RC.

### Survival-associated DEAS events in left- and right-sided colon cancer

**Figure 3:**
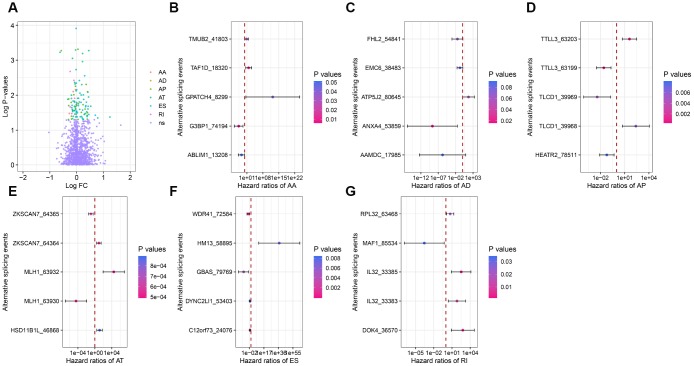
**Forest plots for subgroup analyses of survival-associated DEAS.** (**A**) Volcano plot depicting the P-values from univariate Cox regression analysis of the 1248 DEAS. Log FC: the log-transformed fold-change in the PSI value of a DEAS. (**B**–**G**) Forest plots of hazard ratios for the five AS events with the smallest P-values in the AA, AD, AP, AT, ES and RI splicing patterns, respectively. P-values are indicated by the color scale on the side. Horizontal bars represent the 95% confidence intervals.

To investigate the relationship between DEAS and overall survival in colon cancer patients, we performed a univariate Cox regression analysis of the 1248 DEAS events in the 434 patients. As shown in Figure 3A, 114 survival-associated DEAS were identified (P<0.05). Six of the seven splicing patterns (all but the ME pattern) contained survival-associated DEAS. The AT pattern contained the most survival-associated DEAS (48 cases), followed by the ES pattern (31 cases). The AD pattern contained the fewest survival-associated DEAS (2 cases). For each splicing pattern, the hazard ratios of the five AS events with the smallest P-values are visualized in Figure 3B–3G.

**Figure 4:**
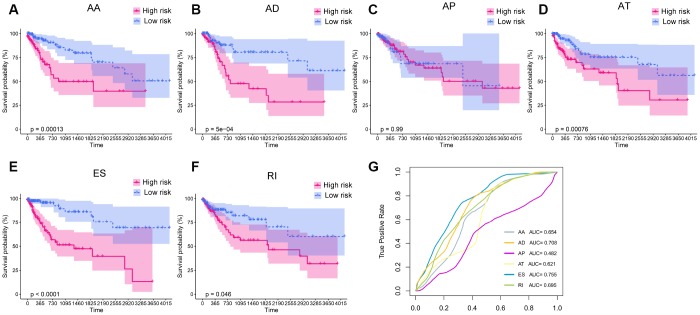
**Kaplan-Meier plots and ROC curves of prognostic models for different AS patterns.** (**A**–**F**) Kaplan-Meier curves of prognostic models built with the AA, AD, AP, AT, ES and RI patterns of AS, respectively. The red line indicates the high-risk group, whereas the blue line indicates the low-risk group. (**G**) The ROC curves of the predictive models for the different AS patterns.

Next, we sought to identify independent prognostic DEAS in colon cancer. Since the univariate Cox regression was only a preliminary screening, we used a relatively loose filter (P-value <0.15) to select variables for multivariate Cox regression analysis. We performed separate multivariate Cox regression analyses for the six splicing patterns. The multivariate Cox regression analysis results for each pattern of AS events are shown in Figure 4A–4F. Colon cancer patients were divided into high-risk and low-risk groups according to the median risk scores predicted by the prognostic models. Five of the six prognostic models exhibited significant power to distinguish good from poor outcomes in colon cancer patients. The prognostic model based on the ES pattern was the most powerful, with a P-value <0.0001. To further assess the discriminatory abilities of these prognostic models, we generated receiver operating characteristic (ROC) curves and calculated the area under the curve (AUC) (Figure 4G). The prognostic model based on the ES pattern displayed the greatest discriminatory ability, with an AUC of 0.755.

**Figure 5:**
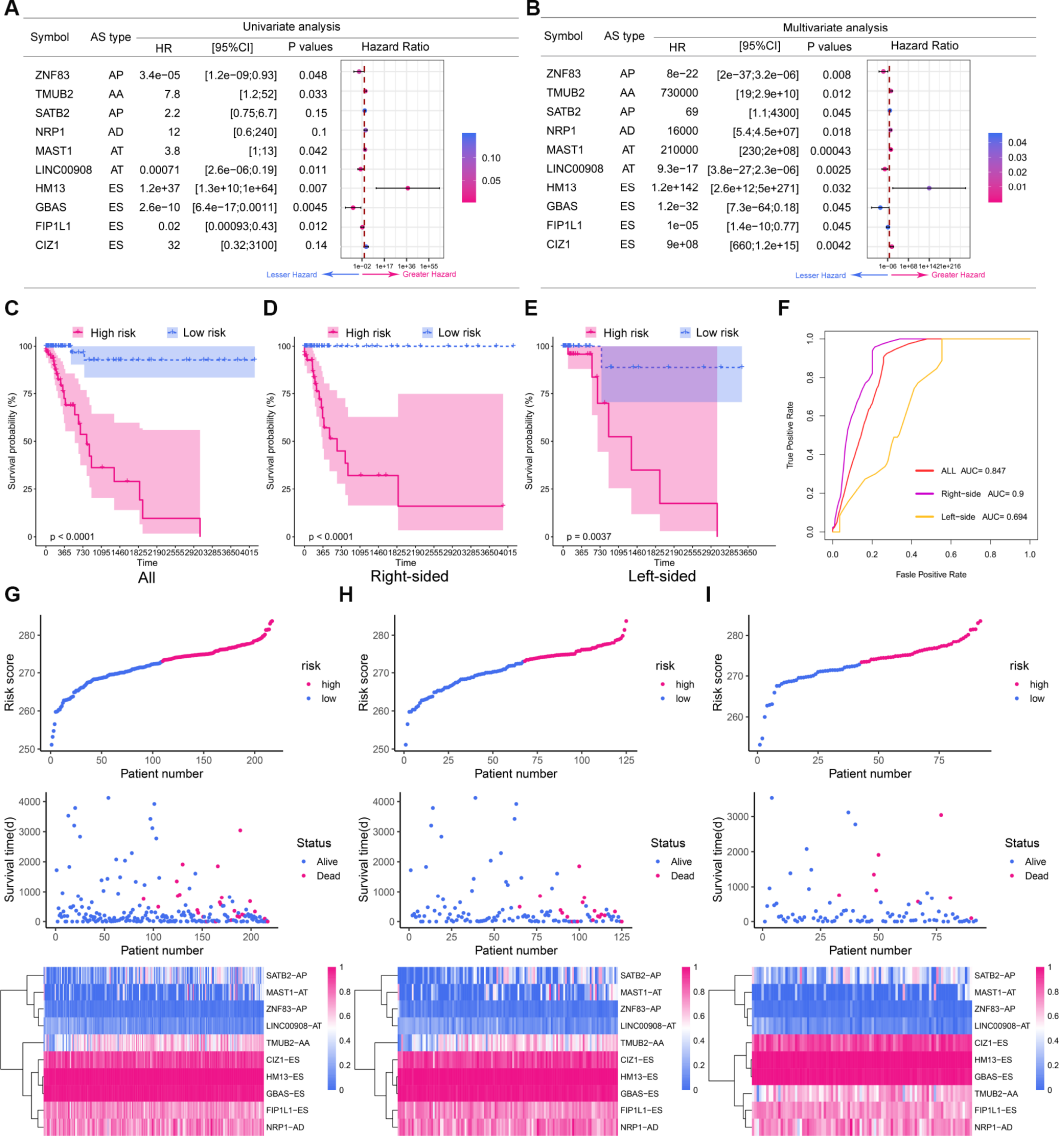
**The prognostic value of the DEAS signature.** (**A**) Univariate analysis of the 10 predictive factors for overall survival. P-values are indicated by the color scale on the side. Horizontal bars represent 95% confidence intervals. (**B**) Multivariate analysis of the 10 predictive factors for overall survival. P-values are indicated by the color scale on the side. Horizontal bars represent 95% confidence intervals. (**C**) Kaplan-Meier curves of the final prognostic model. Patients were divided into the high-risk and low-risk groups according to the median risk score. (**D**) Kaplan-Meier curves of the final prognostic model in right-sided colon cancer patients. (**E**) Kaplan-Meier curves of the final prognostic model in left-sided colon cancer patients. (**F**) The ROC curves of the final prognostic model in all, right-sided and left-sided colon cancer patients. (**G**–**I**) Construction and analysis of risk scores. The top panels indicate the risk scores of the patients. The middle panels depict the survival statuses and survival times of the patients distributed by risk score. The bottom panels display the heatmap of the PSI values for the 10 predictive factors distributed by risk score. (**G**) all patients; (**H**) right-sided colon cancer patients; (**I**) left-sided colon cancer patients.

**Figure 6:**
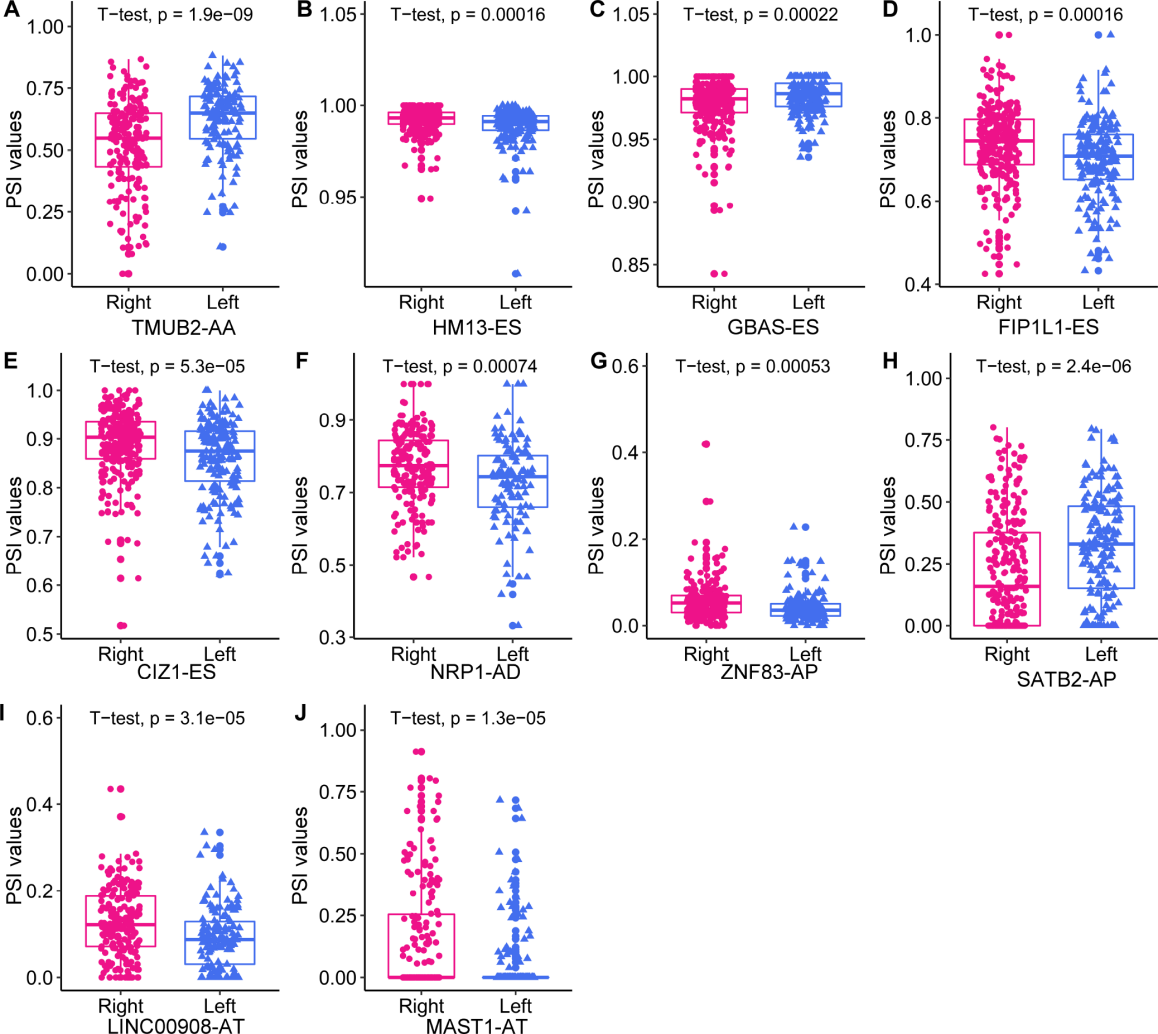
**The expression of the 10 independent prognostic DEAS events in left- and right-sided colon cancer.**

To obtain the final prognostic model, we selected independent prognostic DEAS events from the multivariate Cox regression analysis of each splicing pattern, and further assessed them by multivariate Cox regression analysis. Ten independent prognostic DEAS were selected, and their hazard ratios and P-values are summarized in Figure 5A and 5B. Colon cancer patients were divided into high-risk and low-risk groups according to the median risk score predicted by the final prognostic model. Survival analysis demonstrated that the final prognostic model had significant power to distinguish good from poor outcomes in colon cancer patients (P<0.001) (Figure 5C and 5G). Subgroup analysis indicated that the final prognostic model could efficiently distinguish good from poor outcomes in patients with either LC or RC (RC: Figure 5D and 5H; LC: Figure 5E and 5I). ROC curve analysis revealed that the final prognostic model was more efficient than any of the individual splicing-pattern-based prognostic models in distinguishing good from poor outcomes in colon cancer patients (AUC for the final prognostic model: 0.84). The final prognostic model exhibited greater accuracy in RC than in LC (AUC = 0.90 for RC; Figure 5F). Figure 6 displays the expression of the 10 independent prognostic DEAS in LC and RC. Detailed information on the 10 AS events in the prognostic model is provided in Supplementary Table 1.

### Survival-associated DEAS events expression in colon cancer and protein structure prediction

**Figure 7:**
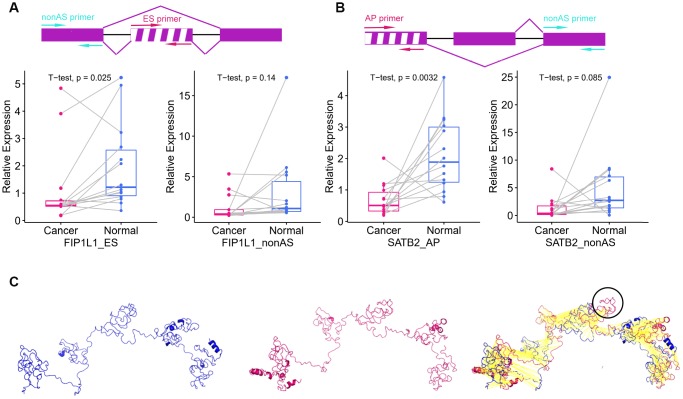
**Survival-associated DEAS events expression in colon cancer and protein structure prediction.** (**A**) The schematic diagram (top panel) depicts the ES of *FIP1L1*, where exon sequences are denoted by boxes and intron sequences are denoted by a horizontal line. The excluded exon is marked with a white stripe. The pair of red arrows indicates the primers amplifying the excluded exon, while the pair of cyan arrows indicates the primers amplifying the common exon among the different isoforms. The left panel displays the expression of *FIP1L1*-ES in cancer and adjacent tissues. The right panel displays the expression of *FIP1L1* in cancer and adjacent tissues. (**B**) The schematic diagram (top panel) depicts the AP of *SATB2*, where exon sequences are denoted by boxes and intron sequences are denoted by a horizontal line. The excluded exon is marked with a white stripe. The pair of red arrows indicates the primers amplifying the excluded exon, while the pair of cyan arrows indicates the primers amplifying the common exon among the different isoforms. The left panel displays the expression of *SATB2*-AP in cancer and adjacent tissues. The right panel displays the expression of *SATB2* in cancer and adjacent tissues. (**C**) Predicted structures of *FIP1L1*. The shorter variant (left panel) and longer variant (middle panel) were predicted by I-TASSER. The black circle in the right panel indicates the structure that could not be aligned.

To explore survival-associated DEAS events expression in colon cancer, we next used reverse-transcription quantitative PCR (RT-qPCR) to assess the expression of three independent prognostic AS events in clinical specimens. Among the three selected AS events, two (*FIP1L1*-ES and *SATB2*-AP) were members of the final prognostic model, while *SMAGP*-AP were independent prognostic AS events from AP pattern models. We designed two pairs of primers to quantify each AS event. One pair of primers specifically amplified the included fragment in the isoform of interest, and was used to quantify the expression of a specific AS product. The other pair of primers amplified the common fragment among the different isoforms, and was used to quantify the total expression of the various isoforms. The expression of *SMAGP*-AP did not differ significantly between cancer samples and adjacent tissues (Supplementary Figure 4). On the other hand, as shown in Figure 7A and 7B, *FIP1L1*-ES and *SATB2*-AP were significantly downregulated in colon cancer samples compared with adjacent tissues (P<0.05). Notably, the expression of the common fragment did not differ significantly between the cancer samples and adjacent tissues, indicating that the differences in AS events between cancer samples and adjacent tissues were not caused by expression changes at the whole-gene level.

Different isoforms of mRNA can be translated into proteins with different structures, thus increasing the diversity of the proteome and the complexity of cells. Therefore, we explored the effects of AS on protein structures. The AP of *SATB2* is located in the 5′ untranslated region, where it can influence the translation process, but not the protein structure. Thus, we only used the ES of *FIP1L1* as an example. The ES of *FIP1L1* occurred in the 11^th^ exon and resulted in two isoforms of *FIP1L1*. The longer variant could be translated into a sequence of 588 amino acids, while the shorter variant could be translated into a sequence of 552 amino acids. The predicated three-dimensional structures of the two variants are shown in Figure 7C. The shorter variant lacked a structure that could have altered the protein functional domain and protein-protein interactions.

### Differentially expressed SFs in left- and right-sided colon cancer

SFs are vital regulators of AS events. SFs bind to pre-mRNAs at specific positions and subsequently process them into mature RNAs. A single SF can induce numerous AS events, and it has been suggested that a limited number of SFs orchestrate the dysregulated AS events in the tumor microenvironment [32]. Given the marked differences in AS events between LC and RC (as illustrated above), we considered it important to explore the relationship between AS events and SFs in LC and RC.

**Figure 8:**
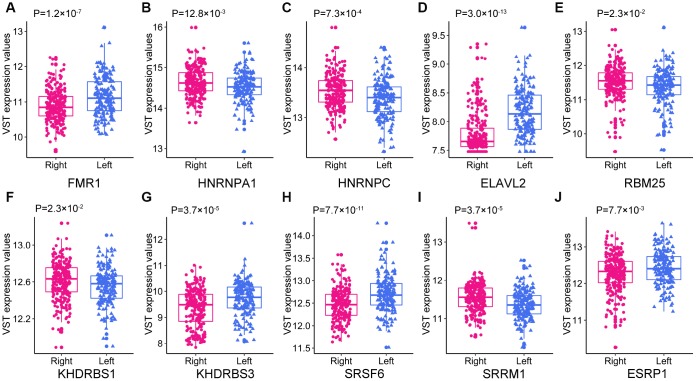
**The expression of the 10 DESFs in left- and right-sided colon cancer.**

First, we identified differentially expressed SFs (DESFs) in LC and RC. The mRNA levels of 71 experimentally validated SFs were obtained and used for differential expression analysis. We identified 10 DESFs in LC and RC. As shown in Figure 8, five SFs were upregulated in RC and five SFs were upregulated in LC. *ELAVL2* exhibited the most significant difference in expression between RC and LC.

### Correlation network of DESFs and DEAS evens

**Figure 9:**
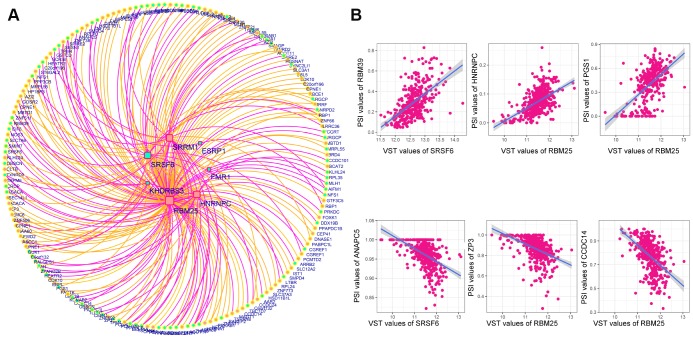
**Correlation network of DESFs and DEAS events.** (**A**) Correlation network. The correlations between the mRNA levels of the 10 DESFs and the PSI values of each DEAS events were analyzed, and a splicing regulatory network was built among the significant correlations. Quadrate nodes indicate SFs that were upregulated in right-sided (red nodes) or left-sided colon cancer (green nodes). Circular nodes indicate AS that were upregulated in right-sided (red nodes) or left-sided colon cancer (green nodes). Orange lines indicate positive correlations, while deep pink lines indicate negative correlations. (**B**) Representative dot plots of correlations between the mRNA levels of DESFs and the PSI values of DEAS (P<0.05).

Next, we analyzed the correlations between the DESFs and DEAS events, and constructed a correlation network from the significantly related pairs (|R| > 0.4 and adjusted P-value <0.05). As shown in Figure 9A, 218 DEAS events were significantly associated with 7 DESFs. Among the 218 DEAS events, 119 were upregulated in RC (orange dots) and 99 were upregulated in LC (green dots). *RBM25* was a hub SF in the correlation network, and was significantly associated with 121 of the 218 DEAS, indicating that it was a key determinant of the distinct AS events in LC and RC. Representative correlations between DESFs and DEAS events are presented as dot plots (Figure 9B). For example, *SRSF6* expression correlated positively with *RBM39* expression, suggesting that *RBM39* could be a potential target of SRSF6.

We then analyzed the clinical significance of *RBM25* in colon cancer. *RBM25* expression was significantly greater in colon cancer tissues than in adjacent tissues (P=2.4×10^-8^). To assess the diagnostic value of *RBM25* in colon cancer, we used an ROC curve to analyze its sensitivity and specificity. The AUC of *RBM25* was 0.672, and its sensitivity and specificity values at a cut-off value of 6.28 variance stabilizing transformation (VST) value were 0.66 and 0.71, respectively. However, a survival analysis indicated that the overall survival did not differ significantly between patients with high and low *RBM25* expression (Supplementary Figure 5).

## DISCUSSION

LC and RC are distinct diseases with highly heterogeneous pathogeneses, molecular characteristics, incidences and prognoses, partly arising from the different embryonic origins of the left and right sides of the colon. Thus, LC and RC are treated by different strategies. Although some of the genomic and epigenetic differences underlying LC and RC have been revealed, much remains unknown.

AS is the main mechanism that accounts for proteome diversity and cell complexity. Aberrant AS is a widely accepted contributor to cancer initiation and maintenance. Several specific AS events in CRC have been identified [29]; however, given the differences between LC and RC, we considered it important to systematically analyze their distinctions at the level of AS and SFs. In total, 1248 DEAS between LC and RC were identified, among which 114 were associated with overall survival. A prognostic signature including 10 survival-associated DEAS was constructed, and an interaction network of DESFs and DEAS was created to provide functional insight into the AS events in LC and RC.

Different splicing patterns produce diverse isoforms of the same gene. In general, AS patterns can be divided into seven types: AA, AD, AP, AT, ES, ME and RI. ES is the most common AS pattern in vertebrates and invertebrates, accounting for around 30% of all AS events [33]. We observed that ES was also the most abundant splicing pattern in colon cancer (40.2%). Interestingly, the proportions of the various splicing patterns differed substantially between LC and RC [33]. The ES pattern was approximately twice as common in RC as in LC, while the RI pattern was roughly 3.5 times more common in LC than in RC. These results indicated that substantial differences existed in LC and RC at the level of AS pattern.

We then performed an enrichment analysis to evaluate the potential functions and pathways of the DSGs. The ‘colorectal cancer’ pathway was enriched in both LC and RC, indicating that the DEAS contributed to the tumorigenesis and progression of CRC. Distinct pathways were also enriched in LC and RC. In RC, the most significant pathway was axon guidance, which is required for the development of the nervous system. In recent years, axon guidance has been reported to be involved in tumor development and progression [34]. An axon guidance signature was found to be associated with poor overall and relapse-free survival, as well as with metastasis and a positive nodal status in CRC [35]. Semaphorins and their receptors, which are crucial axon guidance factors, have been implicated in the migration of tumor cells [36]. Thus, the AS of genes involved in axon guidance may indicate that this pathway contributes to RC tumorigenesis. On the other hand, we observed that immune-related pathways such as B cell receptor signaling and natural killer cell-mediated cytotoxicity pathways were enriched in LC. LC and RC have distinct immune landscapes; for instance, natural killer cell infiltration was reported to be upregulated and associated with prolonged survival in LC [37]. Our results indicated that immune-related pathways may be involved in the tumorigenesis of LC.

To evaluate whether specific DEAS could be used as indicators of colon cancer prognosis, we built prognostic models based on individual AS patterns. The ES pattern was the most efficient in predicting the survival outcomes of colon cancer patients. By combining the different AS patterns, we were able to construct an ideal prognostic model. The final prognostic model included 10 prognostic factors: *ZNF83*-AP, *TMUB2*-AA, *SATB2*-AP, *NRP1*-AD, *MAST1*-AT, *LINC00908*-AT, *HM13*-ES, *GBAS*-ES, *FIP1L1*-ES and *CIZ1*-ES.

NRP1 is a coreceptor with many ligands (most notably, VEGF and semaphorin) [38], and is known to participate in tumor angiogenesis, axon guidance, tumor migration and invasion [39]. Full-length *NRP1*, which contains 17 exons, is translated into a membrane-bound protein. However, ‘reading through’ into introns of *NRP1* leads to the production of two soluble protein isoforms: s_12_NRP1 and s_11_NRP1. These two isoforms are VEGF antagonists, and thus have the opposite function of full-length NRP1. An *in vitro* study demonstrated that s_12_NRP1 prevented VEGF_165_ from binding to cells expressing NRP1. The overexpression of s_12_NRP1 in a rat prostate cancer model increased the percentage of apoptotic cells and reduced the number of blood vessels [40]. In CRC, *NRP1* expression increased significantly across the adenoma-carcinoma sequence [41]. We found that the AD in 14.2 exons of *NRP1* was associated with overall survival in colon cancer. AS could change the protein structure of NRP1, so future studies are needed to explore the functions of diverse NRP1 isoforms in CRC.

CIZ1 is involved in DNA replication initiation and promotes the G1-S phase transition [42]. CIZ1 was found to be upregulated and associated with shorter survival in colon cancer patients [43]. Numerous mRNA variants causing diverse amino acid residue changes in CIZ1 have been identified in humans and mice [44]. For instance, variant CIZ1ΔE4, resulting from ES of exon 4, was found to be upregulated in Ewing’s tumor cells [45]. In mice, partial ES of *CIZ1* exon 6 was reported to impair testis development [46]. In the present study, partial ES of *CIZ1* exon 6 was associated with survival, and thus may be involved in the development of colon cancer.

SATB2, a transcription factor involved in chromatin remodeling, is known to be downregulated in CRC, and can distinguish CRC from other cancer types with high sensitivity. High expression of SATB2 is associated with a good prognosis [47]. However, little is known about the expression and function of the diverse isoforms of SATB2 in CRC, although AS could be expected to alter the binding sites of SATB2 and thus modify its function.

Since numerous AS events can be induced by only a few critical SFs, we sought to identify DESFs between LC and RC. Five SFs were upregulated in RC, while five SFs were upregulated in LC. We constructed a correlation network to describe the relationships between DESFs and DEAS. Among the 218 DEAS in the network, 121 were significantly associated with *RBM25*, demonstrating that RBM25 is an important contributor to the distinction between LC and RC. Analysis of the topological structure of the network indicated that *RBM25* was a hub SF. RBM25, which belongs to a family of RNA-binding proteins, localizes to the nuclear speckles, where it assembles splicing complexes and splices mRNAs [48]. RBM25 is essential for proliferation in many cell lines [49]. Carlson et al. [49] found that RBM25 promoted the inclusion of at least 20% of AS cassette exons in the human genome, suggesting a global splicing factor role of RBM25. High-throughput sequencing revealed that the knockdown of *RBM25* remarkably altered the transcriptome, especially genes encoding proteins involved in metabolic processes and mitochondrial components. We speculate that RBM25 widely orchestrates gene expression throughout the genome, while mainly influencing cellular metabolism. However, the function of RBM25 in CRC remains unclear, and further studies are needed to explore the specificity and mechanism of RBM25 in processing pre-mRNAs.

Several limitations of our study should be mentioned. First, we used relatively loose criteria to generate our set of AS events (events occurring in ≥75% of samples with an average PSI value ≥0.05). Although these criteria enabled us to identify a large number of potentially important AS events, they may have affected the reliability of our study. Further studies with stricter criteria and molecular biology experiments are needed to validate the results of this study. Second, we only used a small number of tumor samples to validate the DEAS events, so additional studies with larger sample sizes are needed.

In conclusion, to our knowledge, this is the first study to comprehensively analyze the differences in AS events and SFs between LC and RC. Prognosis-associated DEAS events were identified, and an interaction network of DESFs and DEAS events was constructed. This study has enriched our understanding of the distinction between LC and RC and provided an extensive list of biomarkers and potential treatment targets for CRC.

## MATERIALS AND METHODS

### Clinical specimens

In total, 14 colon cancer patients who underwent colectomies at the Guangxi Medical University Cancer between June and July of 2019 were included in this study. Colon cancer and adjacent tissue specimens were collected. All patients had primary colon cancer and had not undergone chemotherapy or radiotherapy before the collection of their tissues. The patients included 10 men and 4 women with a mean age of 55.5 years (range: 34–78 years). Detailed clinical information on the 14 patients is shown in Supplementary Table 2.

Written informed consents were obtained from all patients. The study was approved by the Ethics and Human Subject Committee of Guangxi Medical University Cancer Hospital. All experiments and methods were performed according to relevant guidelines and regulations.

### Data acquisition

We downloaded AS data on colon cancer from The Cancer Genome Atlas (TCGA) SpliceSeq, a web-based resource for exploring the AS patterns of 33 different tumor types [50]. PSI values, which range from zero to one, were used to quantify AS events. Given that the PSI values of many AS events were relatively small, we filtered the results (based on ≥75% of samples having an AS event, with an average PSI value ≥0.05) to generate a set of AS events [31]. The level 3 RNA-Seq data and corresponding clinical information from colon cancer patients were downloaded from the Genomic Data Commons data portal (https://portal.gdc.cancer.gov/). The barcodes from TCGA were used to match the AS data, RNA-Seq data and clinical data with each other. Patients who met the following criteria were included in the study: 1. Patients with complete clinical parameters, including sex, age, and information on the cancer location, local invasion, lymph node metastasis, distal metastasis, pathologic stage and survival, and 2. Patients with corresponding RNA-Seq data and AS data. The list of 71 SFs was obtained from SpliceAid-F, a database of experimentally validated SFs [51].

### Identification of DEAS and enrichment analysis

A t-test was performed to identify DEAS events between LC and RC, and P-values were adjusted by the Benjamini-Hochberg method. Given that PSI values are small, we used an adjusted P-value of <0.05 to identify statistically significant DEAS events. The parent genes of these DEAS events were then subjected to GO and Kyoto Encyclopedia of Genes and Genomes (KEGG) enrichment analyses in clusterProfiler [52]. Terms with P-values <0.05 were selected for further analysis. The interactions between the parent genes of these DEAS events were downloaded from the Search Tool for the Retrieval of Interacting Genes/Proteins (STRING) 9.1 database [53]. A required interaction score of 0.9 was used for the protein interaction networks generated in STRING, and the default parameters were used for other settings. Cytoscape v3.4.0 was used to depict the gene interaction network [54].

### Survival analysis

Survival was initially assessed in a univariate Cox regression analysis based on the PSI value of each DEAS product. DEAS events with P-values <0.15 in the univariate Cox regression analysis were entered into the multivariate Cox regression analysis. We first performed multivariate Cox regression analyses based on different AS patterns and constructed corresponding predictive models. Independent DEAS events from the multivariate Cox regression analyses of the different AS patterns were entered into the final multivariate Cox regression analysis. Then, independent DEAS events from the final multivariate Cox regression analysis were used to construct the final prognostic model. Kaplan-Meier curves were used to determine whether the prognostic models could distinguish good from poor outcomes in colon cancer patients. The discriminatory ability of each prognostic model at five years (1825 days) was further assessed by ROC curve analysis in the survival ROC package.

### Identification of DESFs

The count values of the SFs were derived from RNA-seq data. DESFs in LC and RC were identified through the DESeq2 package [55], and the normalized mRNA levels were calculated with the variance-stabilizing transformation function of this program. P-values were adjusted by the Benjamini-Hochberg method. The threshold for DESFs was set at an adjusted P-value <0.05.

### Correlation network construction

The correlations between the normalized mRNA levels of DESFs and the PSI values of DEAS events were calculated with the cor.test function in R. P-values were adjusted by the Benjamini-Hochberg method. Adjusted P-values <0.05 and correlation coefficients with absolute values >0.4 were considered significant. The correlation plots were generated with Cytoscape. The topology structure of the network was analyzed with the NetworkAnalyzer in Cytoscape. A node with over 14 degrees was regarded as a hub node in the network.

### RT-qPCR validation of AS events

RT-qPCR was performed to validate the differential expression of selected AS events. Total RNA was extracted with Trizol reagent (Invitrogen, USA) according to the manufacturer’s instructions. Then, M-MLV Reverse Transcriptase (Promega, USA) was used to reverse-transcribe 2-6 μg of the total RNA into cDNA. RT-qPCR was performed on a qTOWER3 G Real-Time PCR system (Analytik Jena, Germany) in a 20-μL reaction mixture including 0.1 μM primers, 10 μL of GoTaq® qPCR Master Mix (Promega, USA) and 20-100 ng of the cDNA sample. The PCR conditions included denaturing at 95 °C for 10 min, followed by 40 cycles of denaturing at 95 °C for 15 s and annealing and extension at 60 °C for 1 min.

We quantified the expression of specific AS events in a method similar to PSI value calculation, which was the percentage of include exon. We designed two pairs of primers for each gene. One pair specifically amplified the included fragment, and was used to quantify the expression of a specific AS product. The other pair amplified the common exon among different isoforms, and was used to quantify the total expression of the various isoforms. The primers used in the current study are listed in Supplementary Table 3. The relative expression of each gene was calculated by the 2^-ΔΔCT^ method and normalized to that of the reference gene *GAPDH* [56].

### Three-dimensional structural modeling

We employed I-TASSER to predict the three-dimensional structures of different protein isoforms. I-TASSER is a fully automated three-dimensional structure prediction tool that employs a hierarchical approach [57]. The amino acid sequences of different isoforms were entered into I-TASSER, and the three-dimensional structures of the proteins were predicted with the default parameters. PyMol was used for structure visualization, and the ‘align’ function of PyMol was used to compare protein structures.

## Supplementary Material

Supplementary Figures

Supplementary Tables
