# Supplementary material for: Systematic profiling of alternative splicing events and splicing factors in left- and right-sided colon cancer
Source: Aging (Albany NY). 2019 Oct 4;11(19):8270–93. doi: 10.18632/aging.102319 (PMC6814588; doi:10.18632/aging.102319)
Supplement: Supplementary Figures [file aging-11-102319-s002.pdf]

SUPPLEMENTARY FIGURES

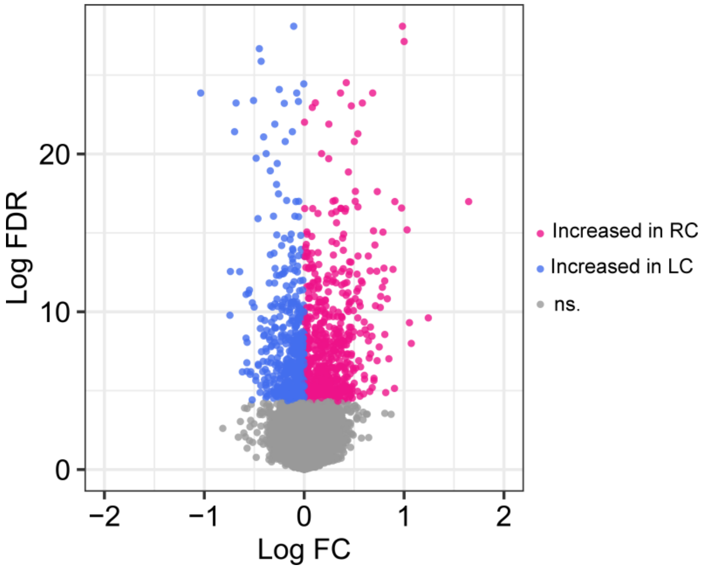

Supplementary Figure 1. Volcano plot of DEAS events. Volcano plot visualizing the DEAS between left- and right-sided colon cancer. The red points represent DEAS that were significantly upregulated in right-sided colon cancer, and the blue points represent DEAS that were significantly upregulated in left-sided colon cancer.

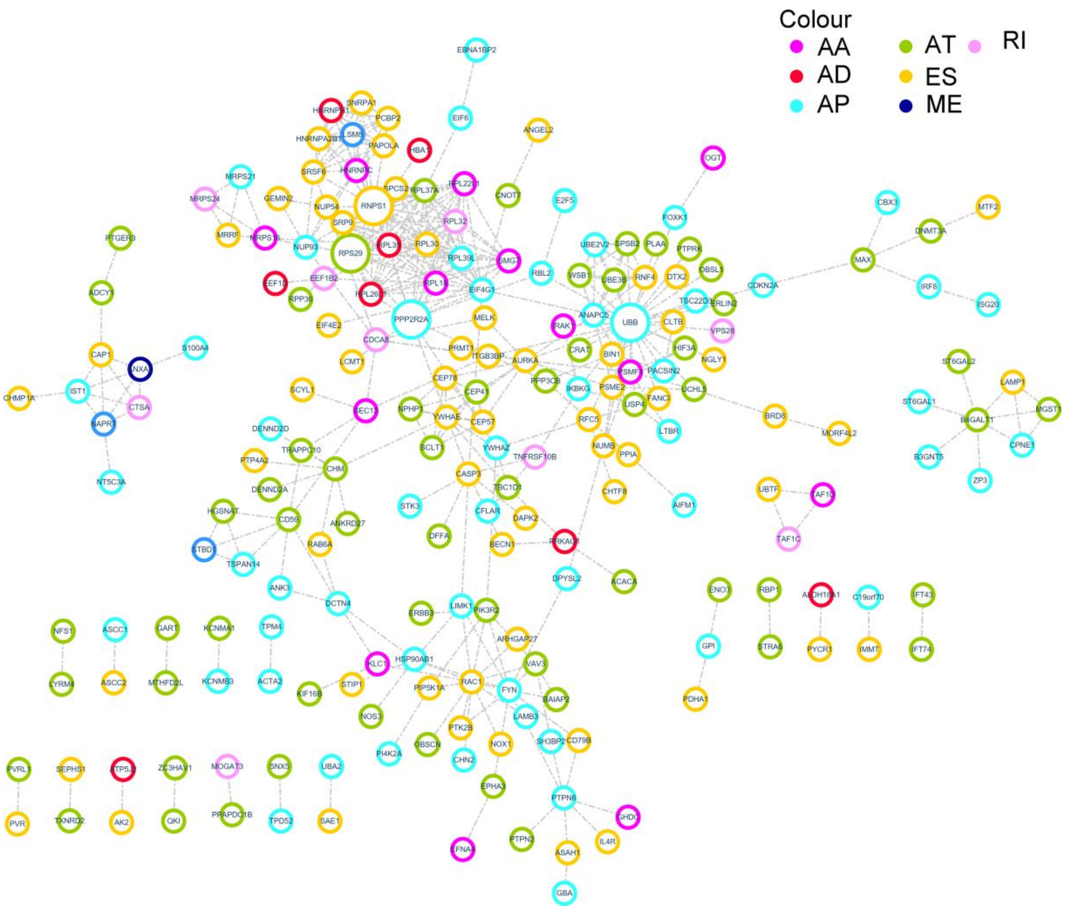

Supplementary Figure 2. Protein-protein interaction analysis of DEAS in left-sided colon cancer. Circular nodes indicate DEAS that were upregulated in left-sided colon cancer. The color of the node represents the AS pattern. Hub nodes in the network are zoomed in.

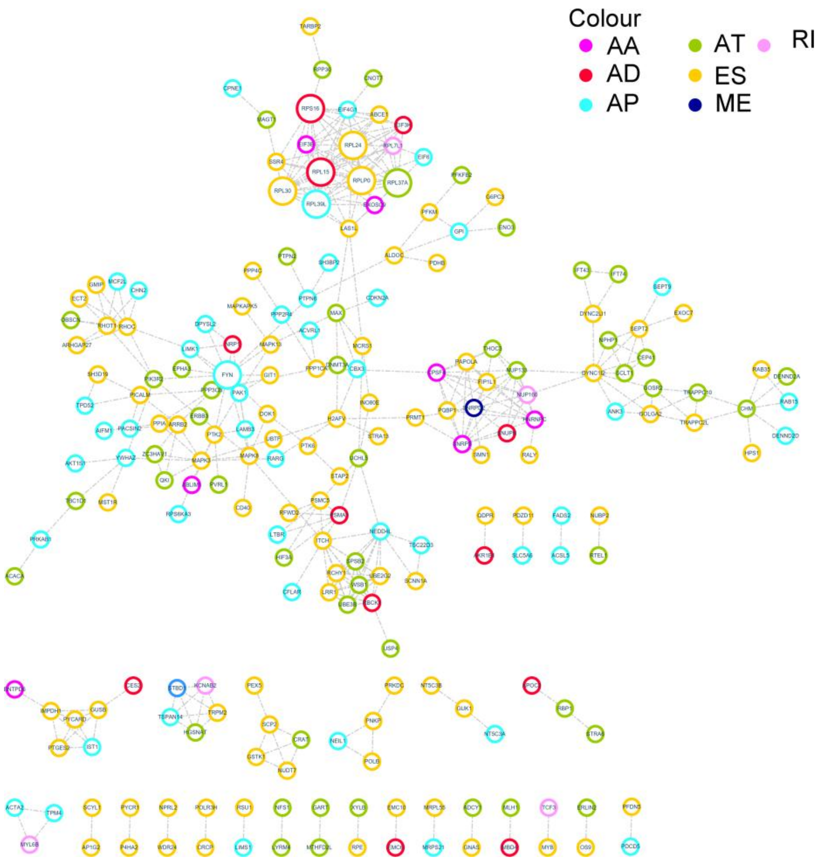

Supplementary Figure 3. Protein-protein interaction analysis of DEAS in right-sided colon cancer. Circular nodes indicate DEAS that were upregulated in right-sided colon cancer. The color of the node represents the AS pattern. Hub nodes in the network are zoomed in.

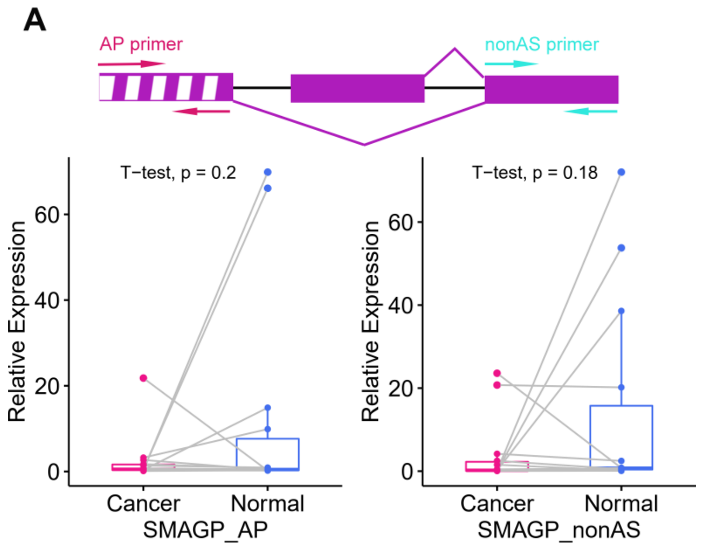

Supplementary Figure 4. The expression of *SMAGP*-AP. (**A**) The schematic diagram (top panel) depicts the AP of *SMAGP,* where exon sequences are denoted by boxes and intron sequences are denoted by a horizontal line. The excluded exon is marked with a white stripe. The pair of red arrows indicates the primers amplifying the excluded exon, while the pair of cyan arrows indicates the primers amplifying the common exon among the different isoforms. The left panel displays the expression of *SMAGP*-AP in cancer and adjacent tissues. The right panel displays the expression of *SMAGP* in cancer and adjacent tissues.

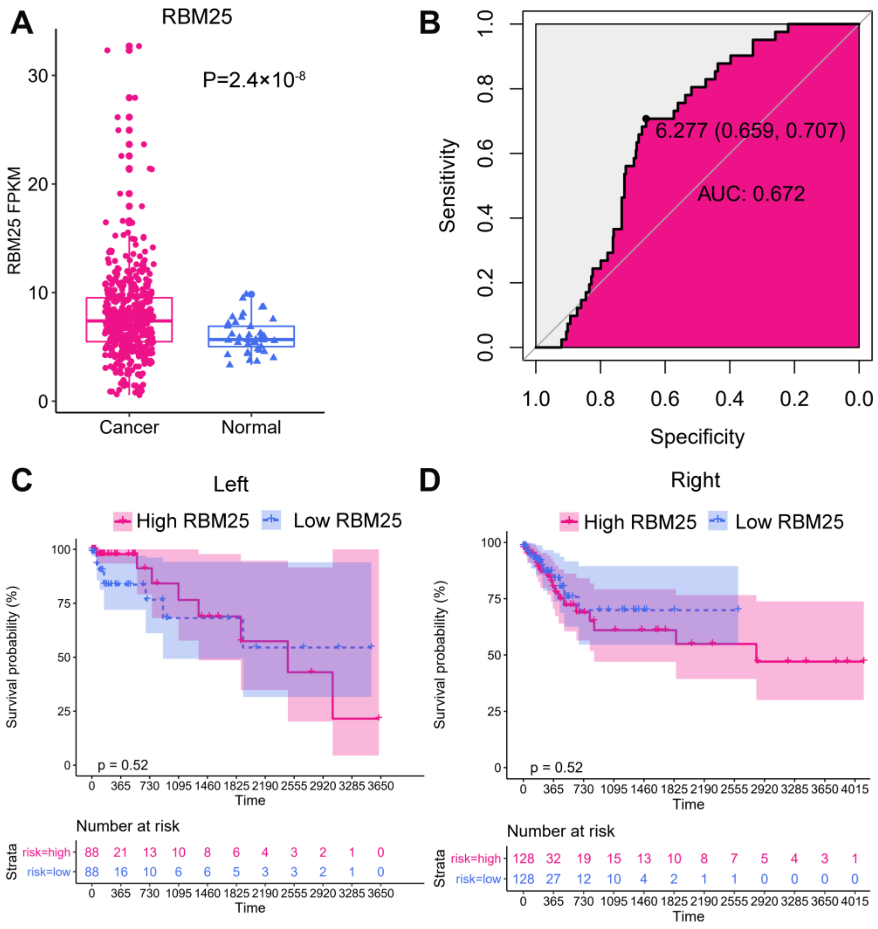

Supplementary Figure 5. The clinical significance of *RBM25* in colon cancer. (**A**) The expression of *RBM25* in cancer and adjacent tissues. (**B**) The ROC curve of *RBM25* in colon cancer. (**C–D**) Kaplan-Meier curves of *RBM25* in left-sided and right-sided colon cancer. The red line indicates patients with high expression of *RBM25*, while the blue line indicates patients with low expression of *RBM25*.
