# Supplementary material for: Systematic profiling of alternative splicing events and splicing factors in left- and right-sided colon cancer
Source: Aging (Albany NY). 2019 Oct 4;11(19):8270–93. doi: 10.18632/aging.102319 (PMC6814588; doi:10.18632/aging.102319)
Supplement: Supplementary Tables [file aging-11-102319-s001.pdf]

SUPPLEMENTARY TABLES

Supplementary Table 1. The detailed information of the 10 AS events in the prognostic model.

Symbol	As id	Splice type	exons	From exon	To exon
MAST1	47878	AT	14.2		
CIZ1	87718	ES	6	5	7
ZNF83	51475	AP	6.1		
NRP1	11200	AD	14.2	14.1	15.1
GBAS	79769	ES	6	5	7
HM13	58895	ES	6	5	7
TMUB2	41803	AA	4.2	3	4.3
FIP1L1	69313	ES	11	10	12
LINC00908	45828	AT	3		
SATB2	56716	AP	1		

Supplementary Table 2. The detailed clinical information of the 14 patients.

ID	Diagnosis	Age	Gender	Tumor location	Histological classification	Tumor size	pT classification	pN classification	M classification	Stage
1	Colon Cancer	66	Male	Ascending colon	Moderately differentiated adenocarcinoma	4.5cm*4cm*1cm	T4a	N1b	M1a	IVA
2	Colon Cancer	78	Male	Colon sigmoideum	Moderately differentiated adenocarcinoma	8cm*8cm*5cm	T4a	N0	M0	IIB
3	Colon Cancer	62	Male	Ascending colon	Moderately differentiated adenocarcinoma	6cm*3cm*1cm	T3	N2b	M0	IIIC
4	Colon Cancer	34	Male	Colon sigmoideum	Poorly differentiated adenocarcinoma	5cm*2.5cm*2.3cm	T3	N0	M0	IIA
5	Colon Cancer	48	Male	Ascending colon	Moderately differentiated adenocarcinoma	4cm*3.5cm*1cm	T2	N1b	M0	IIIA
6	Colon Cancer	45	Female	Transverse colon	Moderately differentiated adenocarcinoma	3cm*2.5cm*1cm	T3	N0	M0	IIA
7	Colon Cancer	72	Female	Hepatic flexure of colon	Poorly differentiated adenocarcinoma	12cm*9.5cm*8cm	T3	N1b	M0	IIIB
8	Colon Cancer	35	Female	Transverse colon	Moderately differentiated adenocarcinoma	5.5cm*5cm*2cm	T4a	N0	M0	IIB
9	Colon Cancer	50	Male	Colon descendens	Mucous adenocarcinoma	5.5cm*4.5cm*3cm	T3	N2b	M0	IIIC
10	Colon Cancer	54	Male	Colon sigmoideum	Moderately differentiated adenocarcinoma	4cm*3cm*1cm	T4a	N1b	M1	IVA
11	Colon Cancer	68	Male	Colon sigmoideum	Mucous adenocarcinoma	6cm*6cm*3.5cm	T3	N2b	M0	IIIC

12	Colon Cancer	55	Male	Colon sigmoideum	Moderately differentiated adenocarcinoma	7cm*3.5cm*1.3cm	T4a	N1b	M0	IIIB
13	Colon Cancer	62	Female	Ascending colon	Moderately differentiated adenocarcinoma	5cm*5cm*2cm	T3	N2b	M0	IIIC
14	Colon Cancer	48	Male	Colon sigmoideum	Moderately differentiated adenocarcinoma	2.5cm*1.7cm*0.5cm	T4a	N2b	M0	IIIC

Supplementary Table 3. The primers used in the study.

Gene symbol	Primers	
FIP1L1-ES	LEFT PRIMER	ACAGCACTTCTTCTCAGTCTCA
	RIGHT PRIMER	ATCAGGTGATTCGGCCCTC
FIP1L1-nonAS	LEFT PRIMER	GCACCTGGAAGCATTAATGGA
	RIGHT PRIMER	ACTTCAAGTCCCATTCGTATCC
SATB2-AP	LEFT PRIMER	ATACCCGGACCCAGGAGAGA
	RIGHT PRIMER	CCTTGATTTCGAAGGCCCCAA
SATB2-nonAS	LEFT PRIMER	TGTCTTTTGTGTCGTGGAGC
	RIGHT PRIMER	GCAGAGCTGTGAGAATACCC
SM AGP-AP	LEFT PRIMER	CAGGAACCGAAACCCGGAG
	RIGHT PRIMER	AAGAGGCAGATCAGCACCCC
SM AGP-nonAS	LEFT PRIMER	ATCCTGGCTAACACGGTGAA
	RIGHT PRIMER	AACCAACTCATCTCCCAGGG
GAPDH	LEFT PRIMER	AAGGTGAAGGTCGGAGTCAA
	RIGHT PRIMER	AATGAAGGGGTCATTGATGG
